# Rather than inducing psychological reactance, requiring vaccination strengthens intentions to vaccinate in US populations

**DOI:** 10.1038/s41598-021-00256-z

**Published:** 2021-10-21

**Authors:** Dolores Albarracin, Haesung Jung, Wen Song, Andy Tan, Jessica Fishman

**Affiliations:** University of Pennsylvania, Philadelphia, USA

**Keywords:** Diseases, Health care

## Abstract

In a survey and three experiments (one preregistered with a nationally representative sample), we examined if vaccination requirements are likely to backfire, as commonly feared. We investigated if relative to encouraging free choice in vaccination, requiring a vaccine weakens or strengthens vaccination intentions, both in general and among individuals with a predisposition to experience psychological reactance. In the four studies, compared to free choice, requirements strengthened vaccination intentions across racial and ethnic groups, across studies, and across levels of trait psychological reactance. The results consistently suggest that fears of a backlash against vaccine mandates may be unfounded and that requirements will promote COVID-19 vaccine uptake in the United States.

Will requiring a behavior create resistance to it or successfully promote it? What are the psychological implications of requiring a behavior like vaccination? In particular, should we require vaccination for COVID-19 (i.e., a vaccine mandate), or should we simply make a recommendation for employees, students, or customers to vaccinate if they so choose?

Two important streams of research make contrasting assumptions about the impact of requiring people to perform a behavior, such as vaccination. In policy and law scholarship, requirements are expected to guide behavior^1–3^. According to this literature, people may simply conform to what is required because they fear social sanctions or view themselves as ethical individuals who follow norms^1,4^. They may also perceive the requirement as beneficial for them and society at large (for available evidence on vaccine mandates, see^5–10^, for research concerning vaccine passports, see^11^).

In contrast to the above literature, research in psychology and other behavioral sciences often views requirements with skepticism^12^. As implied by the theory of psychological reactance^13–16^, people may resist a requirement they perceive as curtailing their freedom^2^. In a review of the literature on COVID-19 vaccination hesitancy, Lin et al.^17^ warned against mandates arguing that prior resistance to mask mandates suggests that vaccine mandates may also elicit reluctance. Specifically, they wrote: “Framing vaccination as a smart, purposeful personal decision, emphasizing individual’s autonomy could yield greater results.” They went on to state that mandates are likely to elicit resistance “even among originally receptive groups”^17^. Other scholars have cautioned that “considerable part of the target group could be annoyed when the act comes into force, consequently showing reactance and opting out of other vaccines that are still voluntary,” concluding that a well-meant mandate could do more harm than good^16^.

The experimental evidence about the impact of vaccination requirements, however, has been limited. A study on mandating a hypothetical children vaccine in an experimental context found no impact on the vaccination intentions of a sample of American parents^12^. A negative effect was only found in an experiment using a computer vaccination game that forced players to vaccinate against their will, which in turn led them to vaccinate less at a later point of the computer game^12^. Thus, we conducted four studies that provide this evidence.

Our first three studies were within-subjects experimental to ensure that intentions to vaccinate varied as a function of requirement and freedom of choice rather than as a result of idiosyncratic characteristics of the participants. The first included a survey of White American, Black American, and Hispanic American respondents that examined the degree to which requiring the COVID-19 vaccine to work, travel, or go to school would increase or decrease intentions to vaccinate. This study was followed by three experiments, the final one preregistered, comparing different scenarios about a vaccination decision in the workplace. Respondents considered the situation in which either a new employer (Studies 2 and 3) or their current employer (Study 4) tells them that (a) “they require that you get vaccinated against a new disease” (requirement condition), (b) “they prefer that you get vaccinated against a new disease but that vaccination is up to you” (freedom of choice condition), and (c) “getting vaccinated against a new disease would give you more freedom and flexibility” (a condition that also emphasizes freedom but controls for freedom of choice, present in Studies 2 and 3).

In each experiment, we examined the impact of the requirement manipulation on vaccination intentions. In Study 4, we also examined the impact of the requirement manipulation on perceived vaccination benefit (i.e., positive outcomes of receiving the vaccine), perceived fairness (i.e., perception that receiving the vaccine is fair to and respectful of individuals), perceived obligation (i.e., perception that vaccinating is moral and law-abiding), and perceived vaccination norms (i.e., perception that vaccination is popular and prevalent). In addition, we examined whether the requirement affected participants with different levels of psychological reactance (Studies 3 and 4), which is the principal reason why requirements could backfire. We also studied whether norms moderated the impact of the requirement (Study 3).

Norms and psychological reactance were important to study because they might be preconditions for negative effects of vaccination requirements. Individuals with higher levels of trait psychological reactance may resent the requirement and thus form weaker intentions to vaccinate, particularly if vaccination norms are weak. The data and codes from our studies are available in https://osf.io/y2rjn/?view_only=1f313bc39b234ed4be0d30c44d8ff126. Each study and its results are summarized below. For additionall information, such as materials and verbatim items, statistical power calculations, and demographic characteristics, please see the Methods section at the end of the manuscript and a summary of the studies in Table 1. All research was carried out in accordance with relevant guidelines and regulations. All experimental protocols were approved by the Institutional Review Board of the University of Illinois and all participants provided informed consent. No participants were under 18.

**Table 1:** Summary of methods: Studies 1–4.

Study	Platform	Design	Description	*N*	Demographic information
Study 1	Prolific	2-Cell (vaccine required vs. control) within-subjects experimental design	Participants answered two questions about their intention to get COVID-19 vaccine	299	*M*_age_ = 32.66, *SD*_age_ = 12.01, Female = 55%
Study 2	Mechanical Turk	3-Cell (vaccine required vs. freedom of choice vs. control for choice freedom) within-subjects experimental design	Participants rotated through 3 conditions in random order and indicated their intention to vaccinate in each condition	359	*M*_age_ = 37.99, *SD*_age_ = 11.10, Female = 50%
Study 3	Mechanical Turk	3 (vaccine required vs. freedom of choice vs. control for choice freedom) × 2 (70% of employees are vaccinated vs. 30% of employees are vaccinated) within-subjects factorial experimental design	Participants rotated through 6 conditions in random order and indicated their intention to vaccinate in each condition. Participants also answered questions about psychological reactance	357	*M*_age_ = 37.48, *SD*_age_ = 10.93, Female = 51%
Study 4 (preregistered)	Qualtrics panels	2 (vaccine required vs. freedom of choice condition) between-subjects factorial experimental design	Participants were randomly assigned to one of the two experimental conditions and indicated their intention to vaccinate. Participants also answered questions about psychological reactance, perceived benefits of vaccination, perceived norms for vaccination, perceived fairness of vaccination, and perceived obligation to vaccinate	606	*M*_age_ = 50.63, *SD*_age_ = 19.23, Female = 51%

## Study 1

Study 1 consisted of a survey of 299 participants who were selected to include Non-Hispanic White (*N* = 108), Black (*N* = 105), or Hispanic (*N* = 96) respondents living in the US recruited from the platform Prolific (www.prolific.com) in January 2021. The survey included two questions: “Will you get the COVID-19 vaccine if it is required to work, travel, or go to school?” (requirement question) and “If you could get the COVID-19 vaccine for free today, would you want to be vaccinated today?” (control question), both answered as either “Yes” or “No.”

Analyses of these questions using a two-sided chi-square test appear in Table 2. As shown, across the board, participants were more willing to vaccinate in response to the required question than the control question. To examine participants’ vaccination intentions across each racial/ethnic group, we excluded participants who were racially/ethnically mixed (*n* = 10), leaving us with 105 white-only, 95 black-only, and 89 hispanic-only participants. The results for each racial/ethnic group were similar to the results for the population as a whole. Eighty six percent of white-only respondents reported intending to vaccinate in response to the required vaccination question but only 69 percent of them reported intending to vaccinate in response to the control vaccination question. Ninety four percent of Hispanic-only respondents reported intending to vaccinate in response to the required vaccination question but only 80 percent of them reported intending to vaccinate in response to the control vaccination question. Eighty percent of Black-only respondents reported intending to vaccinate in response to the required vaccination question but only 56 percent of them reported intending to vaccinate in response to the control vaccination question.

**Table 2:** Responses to requirement and control questions: Study 1.

	Requirement question (%)	Control question (%)	
All participants	86	68	χ^2^_(1)_ = 27.61. *p* < .0001, *d* = 0.64
White-only	86	69	χ^2^_(1)_ = 7.81, *p* = .005, *d* = 0.56
Hispanic-only	94	80	χ^2^_(1)_ = 7.19, *p* = .007, *d* = 0.59
Black-only	80	56	χ^2^_(1)_ = 11.69, *p* = .001, *d* = 0.75

## Study 2

Study 2 was conducted in March 2021 with 359 participants recruited from Mechanical Turk (https://www.mturk.com/) and entailed a 3-cell, within-subjects experimental design in which participants rotated through each condition and conditions were presented in random order. Participants were told that they have a new job and the company tells them that (a) “they require that you get vaccinated against a new disease” (requirement condition), (b) “they prefer that you get vaccinated against a new disease but that vaccination is up to you” (freedom of choice condition), and (c) “getting vaccinated against a new disease would give you more freedom and flexibility” (condition that emphasizes freedom as a vaccination outcome and thus controls for freedom of choice). The dependent measure was the intention to vaccinate, which was measured on a scale from 1 (*extremely unlikely*) to 5 (*extremely likely*).

Analyses of variance of intentions as a function of experimental condition showed that vaccination intentions differed as a function of our experimental manipulation; omnibus *F* (2,716) = 6.98, *p* = .001. Specifically, intentions were significantly stronger in the required vaccination condition (*M* = 3.90, *SE* = 0.07) than either the freedom condition (*M* = 3.72, *S*E = 0.07), *t*(716) = 3.63, *p* = .0009, *M*_diff_ = 0.18, *SD*_diff_ = 0.05, 95% CI[0.06, 0.29], *d* = 0.14, or the control for choice freedom condition (*M* = 3.77, *SE* = 0.07), *t*(716) = 2.59, *p* = .026, *M*_diff_ = 0.13, *SD*_diff_ = 0.05, 95% CI [0.01, 0.24], *d* = 0.10. As a reminder, intentions were measured on a 1 to 5 scale, where 1 = *extremely unlikely*, 2 = *somewhat unlikely,* 3 = *neither likely nor unlikely*, 4 = *somewhat likely*, and 5 = *extremely likely*. Therefore, the requirement moved participants closer to the *likely* point of the scale.

## Study 3

Study 3 was conducted in March 2021 and examined the boundaries of our effect. This study involved 357 participants recruited from Mechanical Turk (https://www.mturk.com/) and entailed a 3 (vaccine requirement: requirement, free, and control for choice freedom) × 2 (norms: 70% of employees are vaccinated versus 30% of employees are vaccinated) within-subjects factorial design. This design ensured that all participants rotated through all conditions and the order of the conditions was random.

With respect to the inclusion of a norm manipulation, social norms (the beliefs about what others do) positively correlate with intention to vaccinate against HPV (Human Papilloma Virus)^18^ and influenza^19^ and social norms interventions successfully promote influenza vaccination behavior among health-care workers^20^. Given that Studies 1 and 2 showed generally positive intentions, one could speculate that those samples could have perceived a favorable vaccination norm as well. Moreover, we wanted to directly observe what might happen when vaccination norms are lower, as such conditions could produce reactance. Thus, we introduced a manipulation of norms to gauge if requirements induce resistance when most of the population fails to vaccinate. We also measured trait psychological reactance^21^ because this personal characteristic could affect the degree to which requirements are effective.

The results from Study 3 appear in Table 3. As before, the experimental conditions differed from each other following the pattern of the earlier experiments, omnibus *F*(2, 710) = 10.57, *p* < .0001. That is, intentions were stronger in the requirement condition than in the freedom of choice condition, *t*(712) = 4.42, *p* < .0001, *M*_diff_ = 0.15, *SD*_diff_ = 0.03, 95% CI[0.07, 0.22], *d* = 0.17, and the control for choice freedom condition, *t*(712) = 3.31, *p* = .003, *M*_diff_ = 0.11, *SD*_diff_ = 0.03, 95% CI[0.03, 0.19], *d* = 0.12. Moreover, the manipulation of norms also had an impact, such that participants who were told that 70 percent of the employees were vaccinated formed stronger intentions to vaccinate than did those who were told that only 30 percent of the employees were vaccinated; *F* (1, 355) = 33.55, *p* < .0001, *M*_diff_ = 0.19, *SD*_diff_ = 0.03, 95% CI [0.13, 0.26], *d* = 0.31. In addition, however, intentions to vaccinate were a function of the interaction between the vaccine requirement manipulation, norms, and psychological reactance, *F*(2, 710) = 3.19, *p* = .04. There was also a significant main effect of psychological reactance (*F*[1,355] = 19.16, *p* < .0001) but no significant interactions between requirement condition and reactance (*F*[2,710] = 0.76, *p* = .466, between norm and reactance (*F*[1,355] = 0.56, *p* = .457), or between requirement condition and norm (*F*[2,710] = 0.15, *p* = .863).

**Table 3:** Effects of requiring vaccination, norms, and psychological reactance on vaccination intentions: Study 3.

Norm manipulation	Required *M* (*SE*)	Freedom of choice *M* (*SE*)	Control for choice freedom *M* (*SE*)
**All participants**			
	3.96^1^	3.85^2^	3.82^2^
**Participants with lower reactance (1 *SD* below the mean)**			
70% vaccinated	4.32^a1^ (0.09)	4.15*b*^2^ (0.10)	4.23^a2^ (0.10)
30% vaccinated	4.12^a^ (0.10)	4.08^a^ (0.10)	4.01^b^ (0.10)
**Participants with higher reactance (1 *SD* above the mean)**			
70% vaccinated	3.80^a1^ (0.10)	3.65^a2^ (0.10)	3.67^a2^ (0.10)
30% vaccinated	3.60^b1^ (0.10)	3.38^b2^ (0.10)	3.50^b12^ (0.10)

Within each estimated level of reactance, different number superscripts indicate significant pairwise differences across the required, freedom, and control for choice freedom conditions, and different letter superscripts indicate significant pairwise differences between the two levels of norms.

Intentions were measured on a 1–5 scale, where 1 = extremely unlikely, 2 = somewhat unlikely, 3 = neither likely nor unlikely, 4 = somewhat likely, and 5 = extremely likely.

To further explore the interaction between the requirement manipulation, norms, and psychological reactance, we examined the interaction between the vaccine requirement and psychological reactance for the lower and higher norm conditions separately. When 70% of the population was vaccinated, requiring the vaccine had an advantage over not requiring it, *F*(2,710) for the simple effect of the requirement manipulation = 7.78, *p* = .0005, regardless of participants’ psychological reactance, *F*(2,710) for the interaction between requirement and psychological reactance = 0.33, *p* = .72. Specifically, in these more positive norm conditions, the requirement condition produced stronger intentions than the freedom condition. *t*(712) = 3.85, *p* = .0004, *M*_diff_ = 0.16, *SD*_diff_ = 0.04, 95% CI[0.06, 0.26], *d* = 0.14, and the control for choice freedom condition, *t*(712) = 2.70, *p* = .02, *M*_diff_ = 0.11, *SD*_diff_ = 0.04, 95% CI[0.01, 0.21], *d* = 0.10.

Now, when only 30% of the population supported vaccination, requiring the vaccine also had an advantage over not requiring it, *F*(2,710) for the simple effect of the requirement manipulation = 5.02, *p* = .007, but this effect differed for participants with lower and higher levels of psychological reactance, *F*(2,710) for the interaction between the requirement manipulation and psychological reactance = 3.02, *p* = .049. Specifically, when only 30% of the population supported vaccination and participants had lower reactance (− 1*SD* of the mean), requiring the vaccine had no effect on intentions (requirement vs. control for choice freedom: *t*(1775) = 1.67, *p* = .10; requirement vs. freedom: *t*(1775) = .60, *p* = .55). When only 30% of the population supported vaccination and participants had higher reactance (+ 1*SD* of the mean), the requirement condition produced stronger intentions than the freedom condition *t*(1775) = 3.45, *p* = .0006, *M*_diff_ = 0.23, *SD*_diff_ = 0.07, 95% CI[0.10, 0.35], *d* = 0.16 but was similar to the control for choice freedom condition, *t*(1775) = 1.61, *p* = .11. Overall, we found no evidence that requiring the vaccine undermines vaccination intentions, even when examining its effects across different levels of vaccination norms and psychological reactance.

## Study 4

Study 4 was a preregistered experiment (https://aspredicted.org/blind.php?x=6sb83y) administered to a nationally representative sample of United States adults in April 2021. This experiment involved 606 participants from Qualtrics panels (https://www.qualtrics.com/research-services/online-sample/) recruited with quotas for gender, education, and race/ethnicity, who provided complete data and passed the quality checks instituted by Qualtrics. The sample demographics appear in Table 4, along with the corresponding figures from the 2019 US Census, which were highly comparable.

**Table 4:** Demographic composition of survey respondents in Study 4.

Sample	Study 4	US Census
*N*	606	
% Female	51.3	50.5
**Race/ethnicity**		
% Non-Hispanic White	59	60.3
% Non-Hispanic Black	12.4	13.4
% Hispanic	23.9	18.5
% Asian	3	5.9
% Other (American Indian, Alaska Native, Native Hawaiian, Other Pacific Islander, and two or more races)	6	4.3
**Education**		
% Some high school or less	13.4	10
% High school graduate/GED	26.9	29
% Some college	18	19
% Associate’s/Bachelor’s degree	27.4	30
% Graduate degree	14.4	11

The estimates are from the 2019 US Census.

Participants again considered the requirement and freedom of choice scenarios but in the context of a between-subjects design in which each participant was randomized to either the requirement or the freedom of choice condition. Such a design cannot ensure that all participants are the same but can prevent carry over effects of one condition on another. The study measured vaccination intentions as well as a set of possible mediators including (a) perceived benefits of vaccination, (b) perceived norms for vaccination, (b) perceived fairness of vaccination, and (d) perceived obligation to vaccinate. As in Study 3, we also measured psychological reactance to again explore whether our proposed effects were moderated by this individual difference.

Intentions as well as the four possible mediators of perceived benefits, perceived norms, perceived fairness, and perceived obligation were examined using analysis of co-variance. Because we recruited a nationally representative sample with potentially large demographic and behavioral variability, we included demographic covariates (gender, white race, and employment, coded as 1 = full time, 2 = part time, and 3 = not currently employed) as well as two indicator variables representing receiving the flu shot during the 2020–2021 season and receiving at least one shot of a COVID-19 vaccine. We also included psychological reactance and the interaction between our manipulation and psychological reactance, which was a preregistered exploratory analysis.

The results from this study appear in Table 5, which show again generally favorable vaccination intentions for the study as a whole. As predicted and preregistered, describing vaccination as a requirement led to stronger intentions to vaccinate (*M* = 3.59, *SE* = 0.07) than describing vaccination as a free choice for participants to make (*M* = 3.36, *SE* = 0.07), *F*(1, 593) = 6.24, *p* = .01, *M*_diff_ = 0.23, *SD*_diff_ = 0.09, 95% CI[0.05, 0.42], *d* = 0.10. This effect was present regardless of participants’ level of psychological reactance, *F*(1, 593) for the interaction between requirement and psychological reactance = 0.04, *p* = .85.

**Table 5:** Effects of requiring vaccination and psychological reactance on intentions, perceived benefits, norms, fairness, and obligation: Study 4.

	Requirement	
	Yes	No
**Vaccination intention**		
All participants	3.59 (0.07)	3.36 (0.07)
Participants with lower reactance (− 1*SD* of the mean)	3.70 (0.09)	3.45 (0.10)
Participants with higher reactance (+ 1*SD* of the mean)	3.48 (0.10)	3.26 (0.09)
**Perceived benefits**		
All participants	3.41 (0.04)	3.39 (0.05)
Participants with lower reactance (− 1*SD* of the mean)	3.63 (0.06)	3.44 (0.06)
Participants with higher reactance (+ 1*SD* of the mean)	3.18 (0.06)	3.33 (0.06)
**Perceived norms**		
All participants	3.32 (0.04)	3.31 (0.04)
Participants with lower reactance (− 1*SD* of the mean)	3.47 (0.06)	3.38 (0.06)
Participants with higher reactance (+ 1*SD* of the mean)	3.17 (0.06)	3.24 (0.06)
**Perceived fairness**		
All participants	3.28 (0.06)	3.30 (0.07)
Participants with lower reactance (− 1*SD* of the mean)	3.54 (0.09)	3.43 (0.09)
Participants with higher reactance (+ 1*SD* of the mean)	3.02 (0.09)	3.17 (0.09)
**Perceived obligation**		
All participants	3.28 (0.06)	3.41 (0.06)
Participants with lower reactance (− 1*SD* of the mean)	3.58 (0.08)	3.53 (0.09)
Participants with higher reactance (+ 1*SD* of the mean)	2.98 (0.09)	3.29 (0.08)

Table entries are means and standard errors (parenthetically) obtained from analyses of covariance.

Intentions were measured on a 1–5 scale, where 1 = extremely unlikely, 2 = somewhat unlikely, 3 = neither likely nor unlikely, 4 = somewhat likely, and 5 = extremely likely.

We also analyzed the effects of the experimental manipulation on perceived benefits, perceived norms, perceived fairness, and perceived obligation. The main effects of our manipulation were not significant in any case: *F*(1, 597) = 0.07 for perceived benefits, *F*(1, 597) = 0.01 for perceived norms, *F*(1, 597) = 2.37 for perceived obligation, and *F*(1, 597) = 0.07 for perceived fairness; *p* > .12 in all cases. The effects of our measure of psychological reactance were *F*(1, 597) = 1.74, *ns*, for perceived benefits, *F*(1, 597) = 2.92, *ns* for perceived norms, *F*(1, 597) = 4.25 *p* = .04 for perceived obligation, and *F*(1, 597) = 4.77 *p* = .03 for perceived fairness. The interactions between condition and psychological reactance were significant for perceived benefits, *F*(1, 597) = 6.98, *p* = .01 and perceived obligation *F*(1, 597) = 4.46, *p* = .035, but not for either perceived fairness, *F*(1, 597) = 2.15, *ns*, or perceived norm, *F* (1, 597) = 1.75, *ns*.

In sum, requiring the vaccine continued to strengthen vaccination intentions and did so independently of psychological reactance. Also, requiring the vaccine did not directly influence perceived benefits, perceived norms, perceived fairness, or perceived obligation. However, we were also interested in the possibility that the mediational pathways from the requirement to intentions might differ across levels of psychological reactance, a possibility supported by the finding that the requirement condition interacted with participants’ level of reactance for perceived benefits and perceived obligation, which, as mentioned above, were *F*(1, 597) = 6.98, *p* = .01, for perceived benefits and *F*(1, 597) = 4.46, *p* = .035 for perceived obligation. A decomposition of these interactions showed that when participants had lower (1 *SD* below the mean) reactance, they perceived more benefits in the requirement condition (*M* = 3.63, *SE* = 0.06) than the freedom of choice condition (*M* = 3.44, *SE* = 0.06; *t*(597) = 2.08, *p* = .037, *d* = 0.09. However, their perceived obligation did not differ across the requirement (*M* = 3.58, *SE* = 0.08) and freedom of choice conditions (*M* = 3.53, *SE* = 0.09); *t*(597) = 0.42, *p* = .68. In contrast, when participants were higher (1 *SD* above the mean) in reactance, they perceived similar benefits in the requirement condition (*M* = 3.18, *SE* = 0.06) and the freedom of choice condition (*M* = 3.33, *SE* = 0.06); *t*(597) = 1.65, *p* = .10. However, their perceived obligation was lesser in the requirement condition (*M* = 2.98; *SE* = 0.09) than the freedom of choice one (*M* = 3.29; *SE* = 0.08); *t*(597) =  − 2.56, *p* = .011, *M*_diff_ = 0.31, *SD*_diff_ = 0.12, 95% CI[− 0.54, − 0.07], *d* = 0.10. Mediational analyses to interpret these differences are presented in Supplementary Materials.

## General discussion

Our surveys and experiments consistently suggested that making vaccination a requirement for work, attending school, or travelling is likely to be successful in promoting vaccination, more so than either giving people freedom to choose or reminding them of the freedom the vaccine might confer. Also, we found no evidence that requirements elicited reactance in a way that undermined the likely efficacy of a vaccine mandate. Rather, the behavioral intentions of participants who scored higher in trait psychological reactance were either more positively influenced by the requirement (see Study 3) or as influenced as their lower reactance counterparts (see Study 4). Study 4 also showed that participants who were higher in psychological reactance felt less obligated to vaccinate when the vaccine was required than when it was not. This result is intriguing and suggests that psychological reactance might lead people to feel less morally obligated to follow a mandate but still be motivated to vaccinate. All in all, our results suggest that fears about a mandate backfiring may be unfounded and support vaccine mandates as a strategy for achieving herd immunity for COVID-19 in the United States.

## Methods

### Study 1

#### Participants

In January 2021, participants were recruited from the Prolific platform (prolific.com), with quotas for US Non-Hispanic White (*N* = 108), Black (*N* = 105), and Hispanic (*N* = 96) respondents. A priori power analysis estimated 225 as the minimum sample required to achieve 85% power to detect a small effect (Cohen’s *w* = 0.15) at an alpha of 0.05. Here and across all our studies, we oversampled to ensure more than adequate power. Participants were 32.66 years old in average (*SD* = 12.01), and 55 percent of them were female. The majority of participants had some college (28%) or college or higher education (56%), whereas the rest had some high school or a GED certificate (15%), or less than high school education (1%).

#### Methods and measures

As an introduction to the vaccination questions, participants received some basic information about the vaccine, which had received provisional FDA approval and had begun to be administered in the US. The information read “Is the COVID-19 vaccine safe? Yes, this vaccine is safe and it works very well. It is about 95% effective, which means that almost everyone who gets vaccinated will be protected and not get sick. The safety and efficacy have been studied carefully by those who work independently from the federal government, politicians, and companies making the vaccine. You can’t get COVID-19 from the vaccine because the vaccines do not include any live virus.”

After reading this basic information, participants received two questions: “Will you get the COVID-19 vaccine if it is required to work, travel, or go to school?” and “If you could get the COVID-19 vaccine for free today, would you want to be vaccinated today? They answered each question as either “Yes” or “No.”

### Study 2

#### Participants and design

In March 2021, we recruited 360 participants recruited from Mechanical Turk (https://www.mturk.com/) and entailed a 3-cell, within-subjects experimental design. A priori power analysis estimated 210 as the minimum sample required to achieve 85% power to detect a small effect (Cohen’s *d* = 0.20) at an alpha of 0.05. This design contained 3 conditions (required, preferred, and control for choice freedom) manipulated within subjects, with each participant rotating through each condition presented in random order. Because one participant did not complete all intention measures, the final sample was *N* = 359. Participants were 50 percent female and 37.99 (*SD* = 11.10) years old on average. With respect to race, 79 percent of the respondents were white, 10 percent were black, 11 percent were Asian-American, 1 percent were American Indian or Alaska Natives, and 0.2 percent were Native Hawaiian or Pacific Islander. With respect to ethnicity, 12 percent were Hispanic.

#### Measures

Participants were told that they had a new job and the company told them (a) “they require that you get vaccinated against a new disease” (required condition), (b) “they prefer that you get vaccinated against a new disease but that vaccination is up to you” (preferred condition), and (c) “getting vaccinated against a new disease would give you more freedom and flexibility” (control for choice freedom condition). The conditions consisted of the following information (bold is ours):

You are about to begin a new job! The company tells you that *they require* that you get vaccinated against a new disease. (Required Condition)

You are about to begin a new job! The company tells you that *they prefer* that you get vaccinated against a new disease but that vaccination is up to you (Freedom of Choice Condition)

You are about to begin a new job! The company tells you that getting vaccinated against a new disease will *give you more freedom and flexibility*. (Control for Choice Freedom Condition).

The dependent measure was the intention to vaccinate, which was measured by the question, “How likely are you to get the vaccine?” with responses measured on a scale from 1 (*extremely unlikely*) to 5 (*extremely likely*). Specifically, intentions were measured on a 1 to 5 scale, where 1 = *extremely unlikely*, 2 = *somewhat unlikely,* 3 = *neither likely nor unlikely*, 4 = *somewhat likely*, and 5 = *extremely likely*. This item was analyzed with parametric methods retaining all five points of the scale.

### Study 3

#### Participants and design

In March 2021, we recruited 358 participants from Mechanical Turk (https://www.mturk.com/) and the study involved a 2 (Norms: 70% vs. 30% support vaccine) × 3 (requirement, freedom, and control for choice freedom) within-subjects experimental design. This design contained 6 conditions (required, preferred, and control for choice freedom crossed with 70% and 30% norm supporting the vaccine) manipulated within subjects, with each participant rotating through each condition presented in random order. A priori power analysis estimated 276 as the minimum sample required to achieve 85% power to detect a small effect (Cohen’s *d* = 0.20) at an alpha of 0.05. Because one participant did not complete all intention measures, the sample used in the analyses of intentions was *N* = 357.

Participants were 51 percent female and 37.48 (*SD* = 10.93) years old on average. With respect to race, 77 percent of the respondents were white, 12 percent were black, 12 percent were Asian-American, 0.3 percent were American Indian or Alaska Natives, and 0.3 percent were Native Hawaiian or Pacific Islander. With respect to ethnicity, 12 percent were Hispanic.

#### Procedures and measures

Participants were told to consider a situation in which they have gotten a new job and the company tells them (a) that they require that you get vaccinated against the flu, COVID-19, and tetanus (requirement condition); (b) they prefer that you get vaccinated against the flu, COVID-19, and tetanus but that vaccination is up to you (freedom of choice condition); and (c) that being vaccinated against the flu, COVID-19, and tetanus will give you more freedom and flexibility within the job (control for choice freedom condition). These statements were presented to all participants twice accompanied with the statement that (a) You find out that 70% of their employees are vaccinated (higher norm condition) or (b) You find out that 30% of their employees are vaccinated (lower norm condition). Thus, as explained previously, six statements were randomized within subjects. That is, each participant read each statement, and after each one, participants were asked about their intentions to vaccinate with the question, “How likely are you to get these vaccines?” Responses were provided on a 1 to 5 scale, where 1 = *extremely unlikely*, 2 = *somewhat unlikely,* 3 = *neither likely nor unlikely*, 4 = *somewhat likely*, and 5 = *extremely likely*.This item was analyzed with parametric methods retaining all five points of the scale.

We also measured psychological reactance using a short scale adopted from^21^. The items of the scale are (a) “It disappoints me to see others submitting to standards and rules,” (b) “I become frustrated when I am unable to make free and independent decisions,” (c)“When someone forces me to do something, I feel like doing the opposite,” (d) “I am content only when I am acting of my own free will,” and (e) “It makes me angry when another person is held up as a role model for me to follow.” Responses were provided on a scale from 1 (*does not describe my feelings*) to 5 (*clearly describes my feelings*). The internal consistency of the scale was adequate (Cronbach’s alpha = 0.78). This scale was analyzed with parametric methods retaining all five points of the scale.

### Study 4

#### Participants and design

In April 2021, participants were recruited from Qualtrics panels with nationally representative quotas for gender, education, and ethnicity. A comparison of demographics in our sample and the general US population appears in Table 4.

The design included two cells, one corresponding to the vaccination requirement and the other to freedom of choice. Participants were randomized to each condition of the between-subjects design. In addition to the quotas, Qualtrics ensures that participants proceed at a normal speed, thus removing people who complete the study by allocating less than 9 min in total. Qualtrics also selects out participants who do not pass their attention checks. A total of 606 participants were recruited with these procedures. A priori power analysis estimated 402 as the minimum sample required to achieve 85% power to detect a small effect (Cohen’s *d* = 0.20) at an alpha of 0.05.

Participants were 50.63 years old in average (*SD* = 19.23), and 51 percent of them were female. The majority of participants had at least a high school education (87%), although 13% had less than high school education. Eighteen percent of participants had completed college and 14 percent had at least some postgraduate education. Thus, the sample clearly approximated the US population.

With respect to ethnicity, 59 percent was non-Hispanic white, 12.4 percent Black, 3 percent Asian American 3 percent American Indian or Alaska Native, 0.7 percent Native Hawaiian or Pacific Islander, and 2.5 percent ‘other.’ With respect to ethnicity, 24 percent of the sample was Hispanic. With respect to participants’ flu and covid vaccination status, 56.6 percent of the sample reported having received the flu vaccine, and 54.8 percent of the sample reported having received at least one shot of the COVID-19 vaccine. Lastly, 32.3 percent of the sample was employed full-time, 12.7 percent employed part-time, and 55 percent of the sample was unemployed (this rate included 3.2 percent full-time student, 31.6 percent retired, 8.1 percent unable to work, 12.1 percent other). Given that 126 million US inhabitants were employed full time in April 2021 out of 329.48, the rate of full time employment is similar. Considering that 12.1 percent might have been unemployed, this was similer to the US peak rate of 14.9 percent during the COVID-19 pandemic^22^. Nonetheless, we control for unemployment in our analyses.

#### Procedures and measures

In this study, participants were randomly assigned to one or two scenarios: (a) On your job, the company tells you that they require that you get vaccinated against a new disease (requirement condition), or (b) On your job, the company tells you that you have to make the decision of vaccinating against a new disease for yourself (freedom of choice condition). They were then asked: “How likely are you to get the vaccine?” Their responses were provided on a scale on a 1 to 5 scale, where 1 = *extremely unlikely*, 2 = *somewhat unlikely,* 3 = *neither likely nor unlikely*, 4 = *somewhat likely*, and 5 = *extremely likely*. This item was analyzed with parametric methods retaining all five points of the scale.

In addition, this study measured several potential mediators. Participants were told “When your employer tells you that you have to make the decision of vaccinating against a new disease for yourself, what do you think?” They then rated 18 statements on a scale from 1 (*extremely unlikely*) to 5 (*extremely likely*). These statements were used to create four indexes. An index of *perceived benefits* was created by averaging the following items: (a) “The vaccine will protect me from this new disease,” (b) “The vaccine will protect other people from this new disease,” (c) “The vaccine will allow me to go to work,” (d) “The vaccine will please my coworkers,” (e) “The vaccine will please my employer,” (f) “Getting the vaccine will allow me to fit in better,” and (g) “The vaccine may be risky” (reversed-scored) (Cronbach’s alpha = 0.83). (Of note, items d-f relate to conformity but excluding them did not alter the results.) An index of perceived *norms* was created by averaging the following items: (a) “The majority of potential employees must want this vaccine,” (b) “Most employees are getting this vaccine,” and (c) “The majority of potential employees are probably getting this vaccine” (Cronbach’s alpha = 0.82). (An additional item “The vaccine is not popular” was reversed scored but did not have an acceptable internal consistency with the other norm items. Consequently, this item was not used but none of the results reported changed as a result of this deletion). An index of perceived fairness was built by averaging (a) “Getting the vaccine is fair” and (b) “Getting the vaccine respects me as a person” (inter-item *r* = 0.89). An index of perceived obligation was built by averaging (a) “Getting the vaccine is the moral thing to do” and (b) “Getting the vaccine is being a law-abiding citizen” (inter-item *r* = 0.86). These scales were analyzed with parametric methods retaining all five points of the scale.

### Supplementary Information

Supplementary Information.
