# Author Correction: Rather than inducing psychological reactance, requiring vaccination strengthens intentions to vaccinate in US populations

**DOI:** 10.1038/s41598-023-46703-x

**Published:** 2023-11-07

**Authors:** Dolores Albarracin, Haesung Jung, Wen Song, Andy Tan, Jessica Fishman

**Affiliations:** University of Pennsylvania, Philadelphia, USA

Correction to: *Scientific Reports* 10.1038/s41598-021-00256-z, published online 21 October 2021

The original version of this Article contained an error in Table 3, where the “Freedom of choice *M* (*SE*)” and “Control for choice freedom *M* (*SE*)” values for “70% vaccinated” and “30% vaccinated” under “Participants with higher reactance (1 *SD* above the mean)” were interchanged. The correct and incorrect values appear below.

Incorrect:

**Table:** 

Norm manipulation	Required *M* (*SE*)	Freedom of choice *M* (*SE*)	Control for choice freedom *M* (*SE*)
**Participants with higher reactance (1 ** ***SD*** ** above the mean)**			
70% vaccinated	3.80^a1^ (0.10)	3.67^a2^ (0.10)	3.65^a2^ (0.10)
30% vaccinated	3.60^a1^ (0.10)	3.50^a2^ (0.10)	3.38^a2^ (0.10)

Correct:

**Table:** 

Norm manipulation	Required *M* (*SE*)	Freedom of choice *M* (*SE*)	Control for choice freedom *M* (*SE*)
**Participants with higher reactance (1 ** ***SD*** ** above the mean)**			
70% vaccinated	3.80^a1^ (0.10)	3.65^a2^ (0.10)	3.67^a2^ (0.10)
30% vaccinated	3.60^b1^ (0.10)	3.38^b2^ (0.10)	3.50^b12^ (0.10)

In addition, the legend of Table 3 was incorrect.

“Within each estimated level of reactance, different letter supscripts indicate significant differences across the required, freedom, and control for choice freedom conditions, and different number supscripts indicate significant differences between the two levels of norms.”

now reads:

“Within each estimated level of reactance, different number superscripts indicate significant pairwise differences across the required, freedom, and control for choice freedom conditions, and different letter superscripts indicate significant pairwise differences between the two levels of norms.”

As a result, in the Study 3 section,

“That is, intentions were stronger in the requirement condition than in the freedom of choice condition, *t*(712) = 3.31, *p* = .003, *M*_diff_ = 0.11, *SD*_diff_ = 0.03, 95% CI[0.03, 0.19], *d* = 0.12, and the control for choice freedom condition, *t*(712) = 4.42, *p* < .0001, *M*_diff_ = 0.15, *SD*_diff_ = 0.03, 95% CI[0.07, 0.22], *d* = 0.17.”

now reads:

“That is, intentions were stronger in the requirement condition than in the freedom of choice condition, *t*(712) = 4.42, *p* < .0001, *M*_diff_ = 0.15, *SD*_diff_ = 0.03, 95% CI[0.07, 0.22], *d* = 0.17, and the control for choice freedom condition, *t*(712) = 3.31, *p* = .003, *M*_diff_ = 0.11, *SD*_diff_ = 0.03, 95% CI[0.03, 0.19], *d* = 0.12.”

And,

“Specifically, in these more positive norm conditions, the requirement condition produced stronger intentions than the freedom condition. *t*(712) = 2.70, *p* = .02, *M*_diff_ = 0.11, *SD*_diff_ = 0.04, 95% CI[0.01, 0.21], *d* = 0.10, and the control for choice freedom condition, *t*(712) = 3.85, *p* = .0004, *M*_diff_ = 0.16, *SD*_diff_ = 0.04, 95% CI[0.06, 0.26], *d* = 0.14.”

now reads:

“Specifically, in these more positive norm conditions, the requirement condition produced stronger intentions than the freedom condition. *t*(712) = 3.85, *p* = .0004, *M*_diff_ = 0.16, *SD*_diff_ = 0.04, 95% CI[0.06, 0.26], *d* = 0.14, and the control for choice freedom condition, *t*(712) = 2.70, *p* = .02, *M*_diff_ = 0.11, *SD*_diff_ = 0.04, 95% CI[0.01, 0.21], *d* = 0.10.”

Finally,

“Specifically, when only 30% of the population supported vaccination and participants had lower reactance (− 1*SD* of the mean), requiring the vaccine had no effect on intentions (requirement vs. freedom: *t*(708) = 2.14, *p* = .08; requirement vs. control for choice freedom: *t*(708) = 1.37, *p* = .36). When only 30% of the population supported vaccination and participants had higher reactance (+ 1*SD* of the mean), the requirement condition produced stronger intentions than the control for choice freedom condition *t*(708) = 3.70, *p* = .0007, *M*_diff_ = 0.38, *SD*_diff_ = 0.10, 95% CI[0.14, 0.62], *d* = 0.14 but was similar to the freedom condition, *t*(708) = 1.48, *p* = .30.”

now reads:

“Specifically, when only 30% of the population supported vaccination and participants had lower reactance (− 1*SD* of the mean), requiring the vaccine had no effect on intentions (requirement vs. control for choice freedom: *t*(1775) = 1.67, *p* = .10; requirement vs. freedom: *t*(1775) = .60, *p* = .55). When only 30% of the population supported vaccination and participants had higher reactance (+ 1*SD* of the mean), the requirement condition produced stronger intentions than the freedom condition *t*(1775) = 3.45, *p* = .0006, *M*_diff_ = 0.23, *SD*_diff_ = 0.07, 95% CI[0.10, 0.35], *d* = 0.16 but was similar to the control for choice freedom condition, *t*(1775) = 1.61, *p* = .11.”

The original Article has been corrected.

