# Supplementary material for: Rather than inducing psychological reactance, requiring vaccination strengthens intentions to vaccinate in US populations
Source: Sci Rep. 2021 Oct 21;11:20796. doi: 10.1038/s41598-021-00256-z (PMC8531364; doi:10.1038/s41598-021-00256-z)
Supplement: Supplementary file 1 — Supplementary Information. [file 41598_2021_256_MOESM1_ESM.docx]

**Supplementary Information**

**Mediational Analyses**

The interactions between the requirement manipulation and psychological reactance for perceived benefits and obligation reported in the paper may imply first that participants with low reactance were likely to go along with the requirement because they saw benefits in meeting it. Those results may also suggest that participants with high psychological reactance perceived a lesser sense of obligation when the vaccine was required, and that this decreased obligation either did not prevent them from forming positive vaccination intentions or strengthened their intentions. To explore these possibilities, we conducted moderated mediation analyses to formally test the combined effects of the requirement manipulation and psychological reactance on vaccination intentions through perceived benefits and perceived obligation. As in the earlier analyses, we controlled for participant gender, white race, employment status, having received the flu shot, and having received at least one dose of the COVID-19 vaccine.

The moderated mediation analyses led to several conclusions. First, the mediating role of perceived benefits for the effect of the requirement manipulation on vaccination intention indeed differed across the levels of psychological reactance (indirect effect = −0.125, *p* = 0.02, 95% CI[−0.23, −0.02]). Specifically, as depicted in Figure 1, participants with lower (1*SD* below the mean) psychological reactance perceived greater benefits and were therefore more likely to vaccinate in the requirement condition than the freedom of choice condition (conditional indirect effect = 0.142, *p* = 0.038, 95% CI[0.008, 0.276]). This mediating effect by perceived benefits was not present among participants with higher (1*SD* above the mean) psychological reactance (conditional indirect effect = –.113, *p* = .100, 95% CI[−0.247, 0.022]). Second, with respect to perceived obligation, participants with higher reactance perceived less obligation when the vaccine was required than when it was not and feeling less obligation ironically fostered positive intentions. As depicted in Figure 1, participants with higher (1*SD* above the mean) psychological reactance perceived lesser obligation and were therefore more likely to vaccinate in the requirement condition than the freedom of choice condition (conditional indirect effect = –0.173, *p* = 0.012, 95% CI[–0.308, –0.039]). This mediating effect was not present among participants with lower (1*SD* below the mean) psychological reactance (conditional indirect effect = .028, *p* = .674, 95% CI[−0.103, 0.160]). Last, as in the analysis of covariance, perceived fairness and perceived norms did not influence the effects of the requirement manipulation on vaccination intentions either alone or in combination with reactance (see Figure 2).


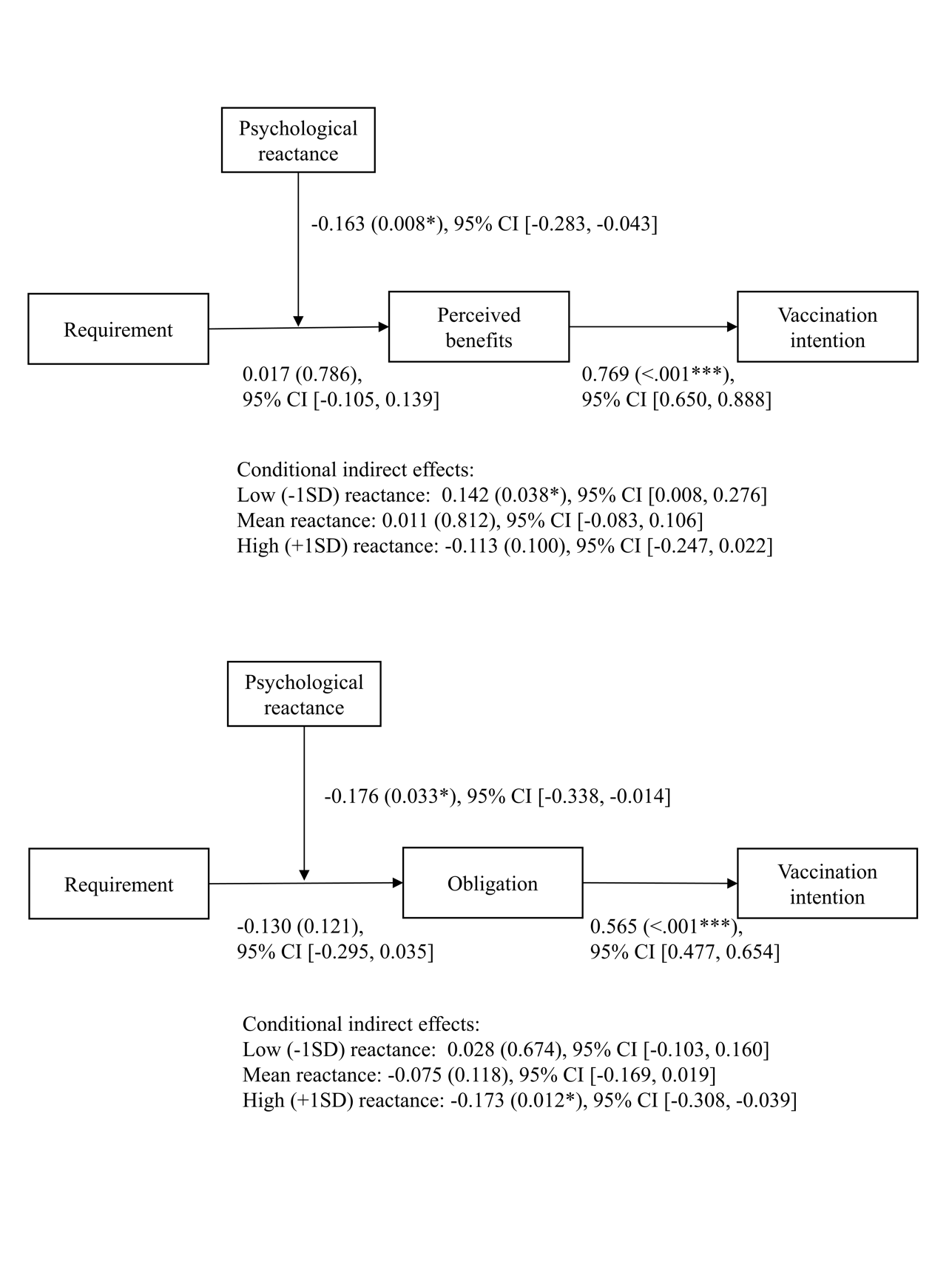


Figure 1. Moderated mediation analyses showing the mediating role of perceived benefits and obligation for the impact of requirement on vaccination intentions as a function of reactance.


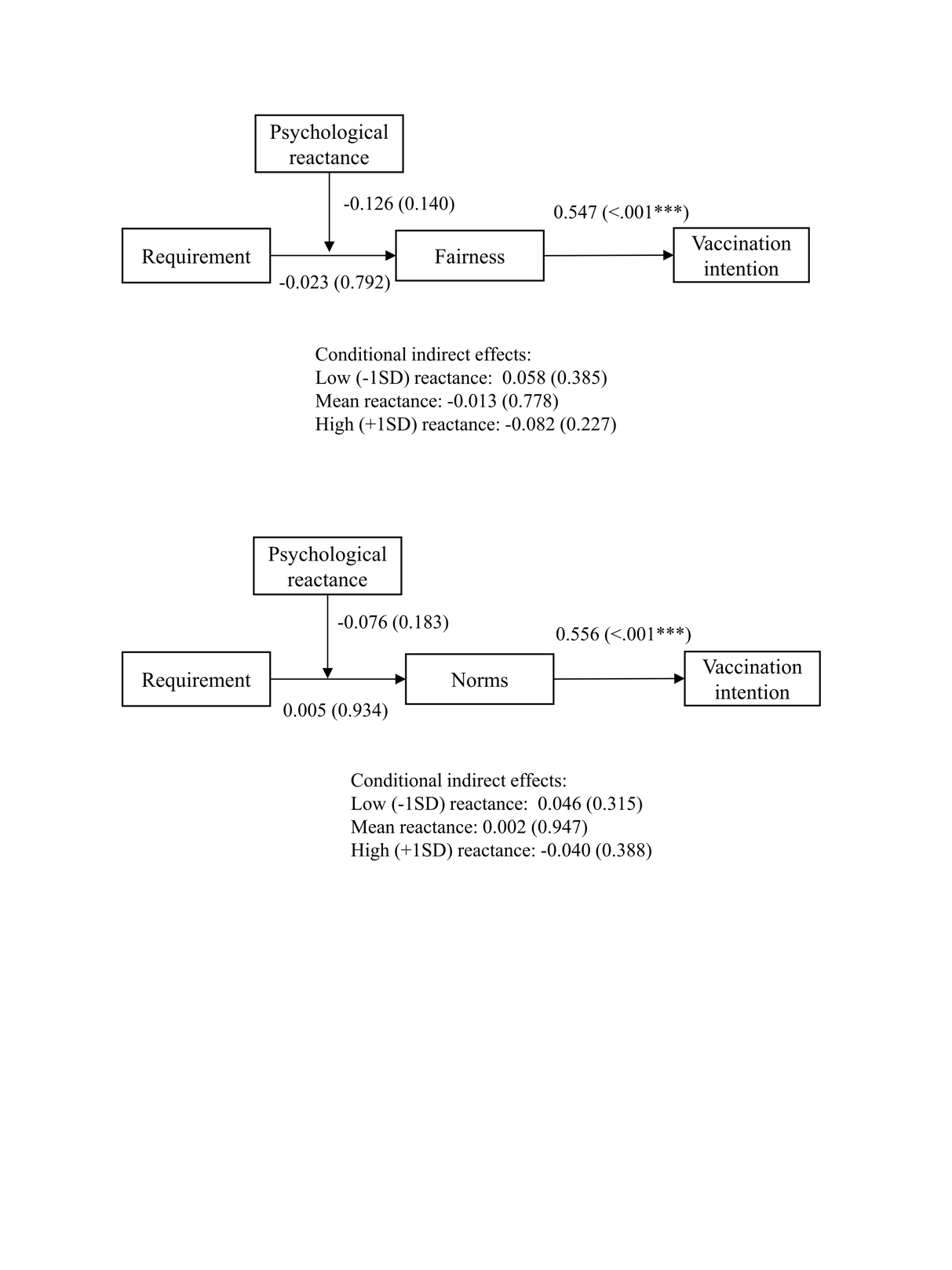


Figure 2. Moderated mediation analyses showing the mediating role of perceived fairness and norms for the impact of requirement on vaccination intentions as a function of psychological reactance.
